# Posture-Induced Intraocular Pressure Changes After Ex-press Implantation Versus Medical Therapy in Primary Open-Angle and Normal-Tension Glaucoma: A Retrospective Study

**DOI:** 10.7759/cureus.94277

**Published:** 2025-10-10

**Authors:** Azusa Yamagishi, Ryusuke Hashimoto, Yuta Kitamura, Takayuki Baba

**Affiliations:** 1 Department of Ophthalmology and Visual Science, Chiba University Graduate School of Medicine, Chiba, JPN

**Keywords:** ex-press, filtering glaucoma surgery, glaucoma, glaucoma drainage device, icare, intraocular pressure fluctuation, intraocular pressure (iop), minimally invasive glaucoma surgery (migs), posture-induced intraocular pressure changes, supine position

## Abstract

Background

Posture-induced intraocular pressure (IOP) changes have been associated with glaucoma progression, and certain filtering surgeries reportedly suppress these changes. This study aimed to determine whether Ex-PRESS implantation also suppresses posture-induced IOP changes in glaucomatous eyes by comparing those treated with Ex-PRESS implantation and those under medical therapy.

Methodology

In this retrospective comparative chart review, 24 eyes after Ex-PRESS implantation without medications (EX group) and 31 eyes that underwent medical therapy (MED group) were analyzed. All eyes were diagnosed with primary open-angle glaucoma or normal-tension glaucoma with IOP measured by Goldmann applanation tonometry (IOPg) between 6 and 12 mmHg. Postural IOP change (ΔIOP) was defined as the difference between supine IOP (IOPisup) measured five minutes after assuming the supine position and sitting IOP (IOPisit), both measured using the iCare ic200.

Results

The IOPg results were comparable between groups (EX: 10.1 ± 1.7 mmHg; MED: 10.7 ± 1.2 mmHg; p = 0.2574). IOPisit was significantly lower in the EX group (EX: 9.1 ± 2.0 mmHg; MED: 10.4 ± 2.2 mmHg; p = 0.0117). IOPisup was also significantly lower in the EX group (EX: 10.2 ± 2.2 mmHg; MED: 13.7 ± 2.6 mmHg; p < 0.0001). Both groups had significant increases from IOPisit to IOPisup (EX: p = 0.0006; MED: p < 0.0001). ΔIOP was significantly smaller in the EX group (EX: 1.0 ± 1.3 mmHg; MED: 3.3 ± 2.2 mmHg; p < 0.0001).

Conclusions

Glaucomatous eyes treated with Ex-PRESS implantation had smaller posture-induced IOP changes than the medically treated eyes with similar IOP levels. These findings suggest that Ex-PRESS may offer additional benefits in stabilizing IOP variability beyond IOP reduction alone.

## Introduction

Glaucoma is a leading cause of blindness worldwide, and high intraocular pressure (IOP) is the most significant risk factor. IOP fluctuates due to factors, including variations during a year [1-3], diurnal changes [4-6], and postural changes [7-10].

Postural IOP changes have frequently been linked to glaucoma [7-9], and several studies have investigated treatment effects on posture-induced IOP changes [11-17]. However, the treatments reported to suppress IOP fluctuations are currently limited to certain filtering surgeries, including trabeculectomy, deep sclerectomy, and XEN [11,14,16-18].

Trabeculectomy remains the gold standard for glaucoma surgery owing to its potent IOP-lowering effects; however, it has a high risk for complications [19]. The Ex-PRESS glaucoma filtration device (Alcon, Fort Worth, TX, USA) is categorized as a filtering surgery similar to trabeculectomy and has produced comparable outcomes with fewer hypotony-related complications [20]. To our knowledge, previous studies have not evaluated IOP fluctuation suppression after Ex-PRESS implantation.

Therefore, we hypothesized that Ex-PRESS implantation, similar to trabeculectomy, may suppress IOP fluctuations. This study compared postural IOP changes between eyes with glaucoma treated with Ex-PRESS implantation (EX group) and those treated with medical therapy (MED group).

## Materials and methods

Ethical considerations

This study adhered to the principles of the Declaration of Helsinki and was approved by the Institutional Review Board (IRB) of Chiba University Hospital (approval number: HK202505-03, with a revised protocol issued on June 13, 2025). As this was a retrospective study, specific informed consent for participation was not required; however, the participants were informed of the study through the protocols posted at the institution, and an opt-out option was made available.

Participants

The eligibility criteria are summarized in Table 1. This retrospective study included patients with primary open-angle glaucoma (POAG) and normal-tension glaucoma (NTG) evaluated for IOP postural changes at Chiba University Hospital between February 2, 2024, and June 6, 2025. We selected (1) eyes that had undergone Ex-PRESS implantation without postoperative glaucoma medications and (2) eyes under medical therapy without prior glaucoma surgery. Eyes with Goldmann applanation tonometer-measured intraocular pressure (IOPg) 6-12 mmHg (following the study by Sawada and Yamamoto [18]) were included; eyes with any history of IOP >21 mmHg were classified as POAG, and eyes with consistent IOP ≤21 mmHg were classified as NTG.

**Table 1:** Eligibility criteria. EX group: Ex-PRESS implantation group; MED group: medical therapy group; IOP: intraocular pressure; IOPg: IOP with Goldmann applanation tonometry; POAG: primary open-angle glaucoma; NTG: normal-tension glaucoma

Eligibility criteria	
Inclusion criteria	
Diagnosis	POAG or NTG (POAG = any history of IOP >21 mmHg; NTG = consistent IOP ≤21 mmHg)
Time window	Evaluated between February 2, 2024, and June 6, 2025, at Chiba University Hospital
IOP range (IOPg)	6–12 mmHg at evaluation
EX group	Eyes after Ex-PRESS implantation without postoperative glaucoma medications at IOP measurement
MED group	Eyes under medical therapy without prior glaucoma surgery
Eye selection	If both eyes were eligible, the right eye was included to avoid inter-eye correlation
Exclusion criteria	
Prior glaucoma surgery	Any prior glaucoma surgery (MED group); surgeries other than Ex-PRESS (EX group). Bleb needling revision was allowed
Coexisting ocular disease	Severe corneal disorders precluding reliable IOP measurement or retinal disorders affecting vision

The exclusion criteria were glaucoma surgeries other than Ex-PRESS (excluding bleb needling revision) and severe corneal or retinal disorders that precluded reliable IOP measurement or affected vision (such disorders were not observed in the patient population). For patients with two eyes that met the inclusion criteria, only the right eye was included to avoid eye correlation bias, which yielded 24 eyes in the EX group and 31 eyes in the MED group.

Measurements

Data collected from patient chart reviews included age, sex, glaucoma type, surgical history, IOPg, sitting/supine IOP (IOPisit/IOPisup) measured using iCare ic200 (Icare Finland Oy, Helsinki, Finland), number of glaucoma medications, best-corrected visual acuity (BCVA), mean deviation (MD) (Central 30-2 Program, Humphrey Visual Field Analyzer, Carl Zeiss Meditec, Inc., Dublin, CA, USA), and central corneal thickness (CCT) (EM-3000, TOMEY Corp., Nagoya, Japan). Preoperative IOP and the number of eye medications used were recorded for the EX group.

The number of glaucoma medications was calculated as follows: each topical antiglaucoma eye drop counted as 1; each fixed-combination eye drop as 2 (drops with ≥3 active ingredients were not used); and oral carbonic anhydrase inhibitors as 2.

IOPg was measured first, followed by IOPisit after ≥5 minutes using the iCare ic200. IOPisup was measured five minutes after placing the patient in a supine position on an ophthalmic surgical bed (Mepro DR-130; Takara Belmont, Osaka, Japan) with the head horizontally aligned. The iCare ic200 was used to obtain triplicate sets of six consecutive readings that were averaged. The IOPg, IOPisit, and IOPisup were measured by the same experienced examiner (AY).

Surgical procedure

In the EX group eyes, a 3 × 3 mm half-thickness scleral flap was created, followed by 0.04% MMC application (three minutes), saline wash, and anterior chamber puncture with a 25 G needle. The Ex-PRESS device was implanted, filtration was confirmed, the flap was sutured with three 10-0 nylon stitches, and the conjunctiva was closed. When combined with phacoemulsification/intraocular lens (IOL), cataract surgery was performed first. Postoperative care included the administration of 0.1% betamethasone (four times a day for three months), 0.5% levofloxacin (four times a day for one month), and 0.5% tropicamide/0.5% phenylephrine (twice a day for one month). Additional interventions (laser suture lysis and needling) were performed when required.

Statistical analyses

The primary outcome was IOPisup minus IOPisit (ΔIOP). All data were de-identified and analyzed using JMP Pro 18 (JMP Statistical Discovery LLC, Cary, NC, USA). Continuous variables were compared using the Mann-Whitney U test; categorical variables were compared using Fisher’s exact test; and paired IOPisit vs. IOPisup were analyzed using the Wilcoxon signed-rank test. A two-sided p-value <0.05 was considered significant. Data are presented as mean ± standard deviation (range) or n (%).

## Results

Table 2 summarizes the patient demographic and clinical characteristics. IOPg was similar in both groups (EX: 10.1 ± 1.7 mmHg; MED: 10.7 ± 1.2 mmHg; p = 0.2574). Additionally, no significant differences were observed for sex or CCT measurements between the EX and MED groups, whereas the EX group was significantly older than the MED group. BCVA and MD were worse in the EX group, which also had a higher POAG-to-NTG ratio. The EX group was also more often pseudophakic. In the EX group, the mean postoperative period was 907.7 ± 578.9 days, the mean preoperative IOP was 21.0 ± 7.6 mmHg, and the mean number of preoperative eye medications was 5.3 ± 1.2.

**Table 2:** Demographic and clinical characteristics. Data are presented as mean ± standard deviation (range) or number (%). ^†^: Applicable to EX only. Significance was set at p-values <0.05. ^a^: Mann–Whitney U test; ^b^: Fisher’s exact test. EX group: Ex-PRESS implantation group; MED group: medical therapy group; IOP: intraocular pressure; IOPg: IOP with Goldmann applanation tonometry; POAG: primary open-angle glaucoma; NTG: normal-tension glaucoma; BCVA: best-corrected visual acuity; HFA: Humphrey field analyzer; MD: mean deviation

	EX group	MED group	P-value
N (eyes)	24	31	-
Age in years	73.8 ± 7.6 (60 to 86)	64.1 ± 10.2 (42 to 82)	0.0007^a^
Sex (women/men)	10/14	17/14	0.4182^b^
IOPg (mmHg)	10.1 ± 1.7 (6 to 12)	10.7 ± 1.2 (8 to 12)	0.2574^a^
Glaucoma type (POAG/NTG), n (%)	16 (66.7)/8 (33.3)	4 (12.9)/27 (87.1)	<0.0001^b^
BCVA (LogMAR)	0.28 ± 0.41 (-0.08 to 1.22)	0.14 ± 0.43 (-0.08 to 1.52)	0.0147^a^
HFA MD (dB)	-20.7 ± 6.5 (-30.1 to -7.68)	-13.0 ± 9.7 (-29.5 to -0.8)	0.0036^a^
Central corneal thickness (μm)	505.5 ± 32.5 (457 to 578)	502.0 ± 31.3 (430 to 545)	0.8007^a^
Lens status (phakic/pseudophakic), n (%)	3 (12.5)/21 (87.5)	25 (80.6)/6 (19.4)	<0.0001^b^
Combined phacoemulsification^†^, n (%)	4 (16.7)	-	-
Postoperative days^†^	907.7 ± 578.9 (248 to 2467)	-	-
Preoperative IOP^†^ (mmHg)	21.0 ± 7.6 (10 to 46)	-	-
Preoperative number of medications^†^	5.3 ± 1.2 (3 to 7)	-	-

Table 3 summarizes posture-related IOP measurements. IOPisit was significantly lower in the EX group (EX: 9.1 ± 2.0 mmHg; MED: 10.4 ± 2.2 mmHg; p = 0.0117). IOPisup increased significantly from IOPisit in both groups (EX: P = 0.0006; MED: p < 0.0001), and it was lower in the EX group (EX: 10.2 ± 2.2 mmHg; MED: 13.7 ± 2.6 mmHg; p < 0.0001). ΔIOP was significantly smaller in the EX group (EX: 1.0 ± 1.3 mmHg; MED: 3.3 ± 2.2 mmHg; p < 0.0001). The mean number of eye medications at the time of measurement was 0 for the EX and 4.0 ± 1.3 for the MED groups.

**Table 3:** Posture-related IOP measurements. Data are presented as mean ± standard deviation (range). Significance was set at p-values <0.05. ^a^: Mann–Whitney U test. EX group: Ex-PRESS implantation group; MED group: medical therapy group; IOP: intraocular pressure; IOPisit: IOP in sitting position with the iCare ic200; IOPisup: IOP in supine position with the iCare ic200; ΔIOP: IOPisup minus IOPisit

	EX group	MED group	P-value
IOPisit (mmHg)	9.1 ± 2.0 (6.1 to 15.6)	10.4 ± 2.2 (5.8 to 15.1)	0.0117^a^
IOPisup (mmHg)	10.2 ± 2.2 (6.6 to 15.4)	13.7 ± 2.6 (9.2 to 20.3)	<0.0001^a^
ΔIOP (mmHg)	1.0 ± 1.3 (-1.1 to 4.1)	3.3 ± 2.2 (-0.8 to 8.5)	<0.0001^a^
Number of medications	0.0 ± 0.0 (0 to 0)	4.0 ± 1.3 (1 to 5)	<0.0001^a^

**Figure 1:**
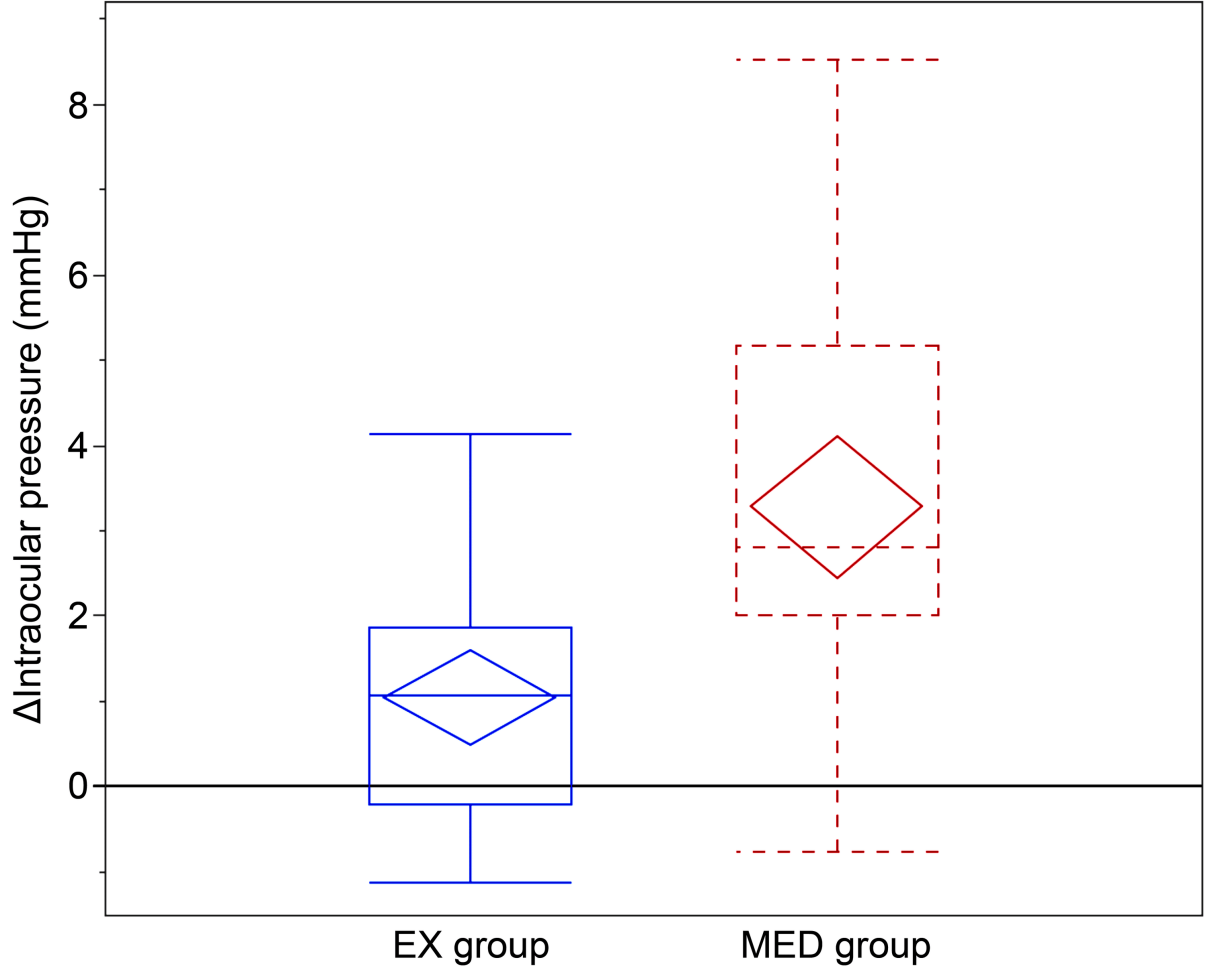
Box-and-whisker plot of postural intraocular pressure (IOP) change (ΔIOP). The box plots represent the median and interquartile range (IQR), whereas the diamonds indicate the mean and 95% confidence interval. ΔIOP was calculated as the difference between IOP in the supine position and IOP in the sitting position. The mean ΔIOP was significantly smaller in the Ex-PRESS (EX) group than in the medical therapy (MED) group (EX: 1.0 ± 1.3 mmHg; MED: 3.3 ± 2.2 mmHg; p < 0.0001). Solid line (blue): EX group; dashed line (red): MED group.

Figure 1 presents a box-and-whisker plot for the distributions of ΔIOP in the EX and MED groups; the EX group had significantly lower ΔIOPs than those of the MED group.

## Discussion

The results of this study demonstrated that glaucomatous eyes treated with Ex-PRESS implantation had significantly smaller postural IOP changes than those that received medical therapy alone, with comparable IOP control.

Recent studies have drawn attention to the relationship between IOP fluctuations and glaucoma progression. The Advanced Glaucoma Intervention Study, Collaborative Initial Glaucoma Treatment Study, and Japanese Archive of Multicentral Databases in Glaucoma reported that long-term IOP changes correlate with visual field deterioration [1,3,21,22]. However, the authors of the Early Manifest Glaucoma Trial and United Kingdom Glaucoma Treatment Study argued that they are not an independent risk factor [2,6]. Similarly, the role of diurnal variation remains controversial; studies support [5] and refute [4,6] its relevance. In contrast, the association between postural IOP changes and glaucoma is widely recognized [4,7-9]. In recent reports, posture-induced IOP changes have been shown to be greater in glaucomatous eyes than in healthy eyes. Specifically, the fluctuations reported are 2.2-3.1 mmHg for healthy eyes [4,23], 3.4-3.8 mmHg for NTG eyes [4,7], and 4.0-4.2 mmHg for POAG eyes [4,23]. Furthermore, larger postural IOP changes are associated with more severe visual field damage [4,7-9].

Postural IOP fluctuations may be particularly important in NTG because, although IOP is well-controlled, fluctuations may still drive progression. In NTG, it has been reported that supine IOP and ΔIOP, but not sitting IOP, were correlated with visual field progression [7], and that postural IOP changes correlated with progression only in NTG eyes among a group that included POAG patients [4]. These findings suggest that fluctuations play a proportionally larger role when the mean IOP is low. The smaller fluctuations observed in the Ex-PRESS eyes than in the medically treated eyes with well-controlled IOP indicate that Ex-PRESS surgery may be particularly useful for patients with NTG and progressive visual field loss despite low IOP.

Our findings are consistent with those of previous reports showing that postural IOP fluctuations are smaller after trabeculectomy than with medical therapy [18]. Most previous studies that assessed the suppression of postural IOP fluctuations compared pre- and post-treatment measurements and did not find a suppression effect from latanoprost, timolol, brinzolamide [24], argon laser trabeculoplasty [12,25], or iStent inject W [13]. In contrast, filtering surgeries, such as trabeculectomy, deep sclerectomy, and even XEN, reportedly reduce postural IOP fluctuation [11,14,16-18]. However, one study did not find a suppressive effect with XEN [15]. Thus, it remains unclear whether all filtering surgeries can reliably suppress IOP fluctuations. Similar to trabeculectomy, Ex-PRESS is a filtering procedure that involves creating a scleral flap and adjusting IOP postoperatively using laser suture lysis. It achieves an IOP-lowering effect comparable to trabeculectomy with fewer hypotony-related complications due to the controlled outflow via the implant [20]. These features suggest that Ex-PRESS offers a similar suppressive effect on IOP fluctuations as trabeculectomy, while potentially minimizing complications.

No significant difference was observed for IOPg between the two groups; however, a significant difference was found for IOPisit. Although IOPg and IOPisit are generally well correlated, the 95% confidence intervals range from -3.4 to 7.33 mmHg [26-28], indicating a degree of variability. Furthermore, reports on the discrepancy between IOPg and IOPisit are inconsistent; one study determined that IOPisit values are lower than IOPg [26], whereas other studies have found the IOPisit values are higher [27,28]. Another study demonstrated that IOPisit tends to have lower values, particularly in eyes with low IOP [26]. Therefore, even a small difference in IOPg between the groups in this study may have become more pronounced in IOPisit values, resulting in statistical significance.

When evaluating postural IOP changes, it is ideal to measure IOP once it stabilizes after a change in position. However, consensus is lacking regarding the required rest period. In the present study, we adopted a five-minute supine rest based on previous studies [11,13,18]. However, other studies have used longer rest periods, such as 10 [12,15] or 30 minutes [17,24], underscoring the need for standardization in the methodology. A previous study that investigated the time course of postural IOP changes found IOP plateaued after 10 minutes [8]; notably, the five-minute timepoint was not evaluated. Moreover, that study excluded eyes with a history of intraocular surgery, indicating that additional research is needed to determine plateau timing in postoperative eyes with glaucoma.

This study has several limitations. First, because of the retrospective nature of the study design, selection bias was possible, and measurement times were not standardized. IOPg measurement preceded that of IOPisit, which potentially lowered IOPisit; however, a ≥5-minute interval before measuring IOPisit likely minimized any carryover. Second, regarding between-group confounding, the EX group was older and had more pseudophakic eyes, a higher POAG-to-NTG ratio, and worse MD. Phacoemulsification can slightly reduce IOP in the short term [29] and may transiently attenuate posture-related fluctuation in healthy eyes [30], although the long-term effects in glaucomatous eyes are unknown. Because fluctuation is age-insensitive [10] and larger in eyes with POAG and with worse MD [4,9], these imbalances would indicate bias against the smaller ΔIOP observed in the EX group. Third, the MED group had heterogeneity, topical regimens were diverse, some agents do not suppress fluctuation [24], and the effects of unexamined agents remain unknown. Fourth, the small sample size limits the robustness of our conclusions. Finally, although the study results suggested fluctuation suppression by Ex-PRESS, preoperative versus postoperative changes were not assessed; thus, generalization of the findings is limited. Prospective studies are warranted.

## Conclusions

This study demonstrated that glaucomatous eyes treated with Ex-PRESS implantation had smaller postural IOP changes than eyes under medical therapy, which indicated a fluctuation-suppressive effect from Ex-PRESS implantation. To our knowledge, this was the first report to describe such an effect from Ex-PRESS implantation and extends existing evidence that filtering surgeries, such as trabeculectomy, can reduce posture-related IOP fluctuations. As postural IOP changes are associated with visual field progression, particularly in NTG eyes, these results suggest that Ex-PRESS implantation may provide additional therapeutic benefits beyond simple IOP reduction. This finding may serve as a guide in surgical decision-making for patients who exhibit disease progression despite low IOP.
